# Evidence for frequent incest in a cooperatively breeding mammal

**DOI:** 10.1098/rsbl.2014.0898

**Published:** 2014-12

**Authors:** H. J. Nichols, M. A. Cant, J. I. Hoffman, J. L. Sanderson

**Affiliations:** 1 Faculty of Science, Liverpool John Moores University, Liverpool L3 3AF, UK; 2 College of Life and Environmental Sciences, University of Exeter, Exeter TR10 9FE, UK; 3 Department of Animal Behaviour, Bielefeld University, Bielefeld 33501, Germany

**Keywords:** inbreeding, incest, cooperative breeding, life-history evolution, reproductive competition, dispersal

## Abstract

As breeding between relatives often results in inbreeding depression, inbreeding avoidance is widespread in the animal kingdom. However, inbreeding avoidance may entail fitness costs. For example, dispersal away from relatives may reduce survival. How these conflicting selection pressures are resolved is challenging to investigate, but theoretical models predict that inbreeding should occur frequently in some systems. Despite this, few studies have found evidence of regular incest in mammals, even in social species where relatives are spatio-temporally clustered and opportunities for inbreeding frequently arise. We used genetic parentage assignments together with relatedness data to quantify inbreeding rates in a wild population of banded mongooses, a cooperatively breeding carnivore. We show that females regularly conceive to close relatives, including fathers and brothers. We suggest that the costs of inbreeding avoidance may sometimes outweigh the benefits, even in cooperatively breeding species where strong within-group incest avoidance is considered to be the norm.

## 1. Introduction

Breeding between close relatives has long been recognized to entail a fitness cost, known as inbreeding depression, which is thought to result mainly from the unmasking of harmful recessive alleles [1]. Consequently, it is not surprising that inbreeding avoidance mechanisms such as dispersal, reproductive restraint and mating with unfamiliar individuals are widespread in the animal kingdom [1]. However, inbreeding avoidance can also entail fitness costs. For example, dispersal is commonly associated with increased mortality [2]. By implication, even inbreeding between first-order relatives should be tolerated under some circumstances [3,4].

Although inbreeding and inbreeding avoidance have fitness consequences in virtually all vertebrates, these effects may be particularly important in cooperative breeders, where natal philopatry can lead to the presence of sexually mature relatives in social groups [5]. Moreover, theoretical work predicts that inbreeding could have a substantial positive effect on inclusive fitness in these species by increasing the reproductive success of relatives [6] and/or increasing the benefits of cooperation [5,7].

Despite these theoretical predictions, evidence that incest forms a regular part of the mating system of mammalian cooperative breeders is scarce and the vast majority of these species appear to have obvious within-group inbreeding avoidance mechanisms [5]. Furthermore, in the handful of species where frequent incest is thought to occur, such as naked mole rats, genetic data are either lacking or insufficient to quantify inbreeding [2,4,5].

Here, we use an unusually large genetic dataset in combination with detailed behavioural observations to investigate inbreeding in the banded mongoose (*Mungos mungo*), a cooperatively breeding carnivore that lives in mixed-sex groups (median group size = 18 adults). Groups consist of a ‘core’ of dominant individuals (one to five females and three to seven males) that reproduce three to four times per year, alongside younger subordinates that breed occasionally. Although some dispersal occurs, many individuals of both sexes remain in the natal group for their entire lives [8]. Both sexes also frequently breed in their natal group, despite the presence of first-order relatives, and there is no evidence of reproductive restraint [9]. Immigration of individuals into established groups is practically absent [8] so opportunities to mate with unrelated immigrants rarely arise. Furthermore, pups are reared in large communal litters, making familiarity an ineffective cue to relatedness [8]. In the absence of any obvious mechanism of within-group inbreeding avoidance, a previous study suggested that inbreeding could be a regular part of the banded mongoose mating system [9].

New banded mongoose groups form when a cohort of female relatives from one natal group joins a cohort of male relatives from a different natal group, resulting in opposite-sex group-members initially being unrelated [8]. However, owing to high levels of philopatry and a lack of immigration, relatedness between opposite-sex breeders builds up over time [10], suggesting that inbreeding could be more prevalent in older groups. Inbreeding might also be more likely to occur when groups are small and choice over mating partners is restricted. Nevertheless, it is also possible that females avoid inbreeding by mating with extra-group males. Although observations of extra-group copulations are rare, neighbouring territories often overlap substantially and groups encounter each other regularly, so opportunities may arise [10].

We use 20 microsatellite markers to assign parentage and to generate a partial pedigree for an intensively studied population of banded mongooses. We quantify the frequency with which females breed within their natal group and test the hypothesis that females mate with close relatives. We also test the predictions that inbreeding is prevalent in older and smaller social groups and that females can avoid inbreeding through dispersal or mating with extra-group males.

## 2. Material and methods

### (a) Behavioural data

We studied a population of 14 banded mongoose groups living in Queen Elizabeth National Park, Uganda (0°12′ S; 29°53′ E) between November 1995 and September 2011. All animals were marked individually and habituated to close observation (less than 5 m). Groups were observed every 1–4 days, allowing individuals to be tracked from birth to death and all dispersal and breeding events to be recorded [8]. Mean adult annual survival in our Ugandan population (females 0.61, males 0.66) is similar to that found in the Serengeti (females 0.69, males 0.65), so it is unlikely that any observed inbreeding is owing to unusually high survival in our study population [11].

### (b) Parentage analysis

A total of 1534 tail tip samples were collected using sterile scissors while animals were anaesthetized. Further details of sample collection and genotyping using 20 microsatellite loci are described elsewhere [10]. Pairwise relatedness was calculated following Lynch & Ritland [12] and parentage was assigned using Cervus [13]. As female group-members usually give birth synchronously, all visibly pregnant females present in the group when a litter was born were considered potential mothers. Owing to the relatively small numbers of candidate mothers (mean = 4.3 per pup), maternities were assigned first. Paternity was then assigned to all pups assigned maternity at 95% confidence or more. Potential fathers included all males in the population over 1 year old at litter conception (approx. 60 days before birth, mean = 72.5 candidate fathers per pup). A total of 629 pups were assigned paternity at 95% confidence or more (90% confidence or more after taking into account the probability of misassigning maternity). For 516 of these pups from 12 groups, the mother's group of birth was known, allowing us to investigate whether dispersal influenced female reproductive behaviour. See the electronic supplementary material for further details on sample sizes. Coefficients of inbreeding were calculated using Pedantics [14] and inbreeding was quantified following [15].

### (c) Statistical analysis

Statistical analyses were conducted in R.3.0.1 using the lme4 package [16]. General linear mixed models (GLMMs) were constructed to test whether inbreeding is more frequent (i) among natal females than dispersed females; (ii) among females that mate with resident rather than extra-group males; and (iii) in older and smaller social groups.

## 3. Results

**Figure 1.**
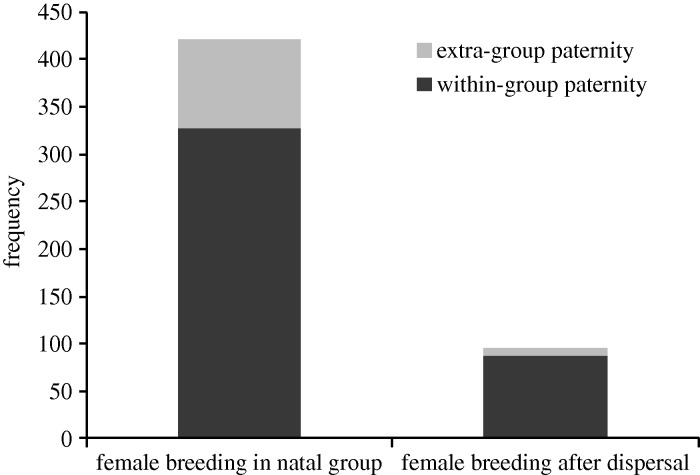
The frequency of within-group and extra-group paternity among the offspring of females breeding (i) in their natal group, and (ii) after dispersal to a new group.

Of a total of 516 pups, 328 (63.6%) were born to females that conceived within their natal group to resident males (figure 1). A further 93 pups (18.0%) were born to females who remained in their natal group but conceived to an extra-group male, and 95 pups (18.4%) were born to females that dispersed out of their natal group (figure 1). A significantly larger proportion of pups were fathered by extra-group males when females stayed within their natal group (93 of 421 pups) in comparison to females that dispersed (8 of 95 pups; binomial proportions test: *χ*^2^ = 8.35, d.f. = 1, *p* = 0.0039), suggesting that natal females may sometimes mate extra-group to avoid inbreeding.

**Figure 2.**
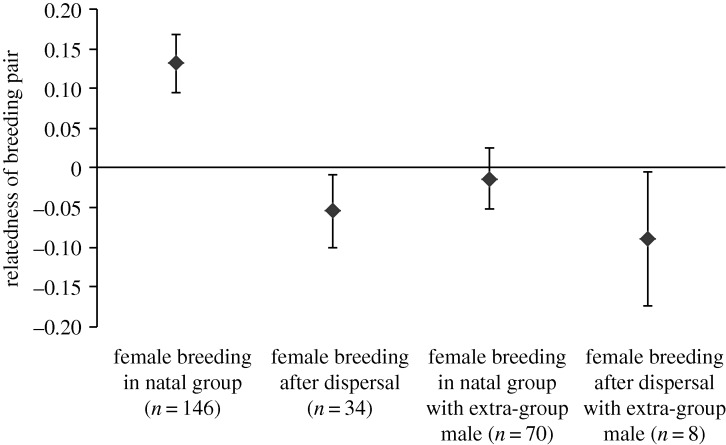
Mean (±95% confidence intervals) relatedness values of banded mongoose breeding pairs depending on whether females bred in their natal group or after dispersal, and with a resident or extra-group male. The 216 breeding pairs included here produced 516 pups.

Relatedness coefficients calculated from microsatellite data [12] revealed that females breeding within their natal group conceived to closer relatives than females that either bred with extra-group males or dispersed (GLMM: 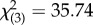, *p* = 8.47 × 10^−08^; figure 2; electronic supplementary material, table S1). A substantial proportion of females that bred within their natal groups conceived to close relatives; 26.71% conceived to a male related by 0.25 or more, and 7.53% conceived to a male related by 0.5 or more. The equivalent proportions for females that did not breed within their natal group were substantially lower, at 4.46% and 0.89%, respectively.

After excluding extra-group paternities, the mean relatedness of parent-pairs increased significantly with group age (GLMM: 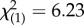, *p* = 0.013; electronic supplementary material, table S2), indicating that inbreeding is more likely to occur in older social groups. There was no evidence for inbreeding being more prevalent in smaller groups (GLMM: 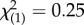, *p* = 0.62; electronic supplementary material, table S2).

Pedigree assignment identified 30 individuals from four social groups with non-zero inbreeding coefficients (*f*). These comprised 11 cases of close inbreeding (*f* = 0.25), seven cases of moderate inbreeding (*f* = 0.125) and 12 cases of weak inbreeding (0 < *f* < 0.125; electronic supplementary material, table S3).

## 4. Discussion

We provide evidence that inbreeding is a regular part of the breeding system of banded mongooses in our study population. The majority of pups were born to females reproducing within their natal groups and, of these, a substantial proportion were conceived to relatives. A high level of inbreeding was also supported by the pedigree data, which revealed close inbreeding (*f* = 0.25) in 8.5% of cases and moderate inbreeding (0.25 < *f* ≥ 0.125) in 16.7% of cases.

Similar rates of moderate inbreeding have been documented in other cooperative mammals, including black tailed prairie dogs (*Cynomys ludovicianus*; 26%, [17]) and meerkats (*Suricata suricatta*; 15%, [18]). However, close inbreeding is far less common and appears to be actively avoided in almost all species [5]. The unusually high rate of close inbreeding in the banded mongoose could be a consequence of group structure, as we found that inbreeding was more common in older social groups. This is probably owing to natal philopatry leading to an increasing encounter rate between opposite-sex relatives over time since groups formed [10].

While all group members could potentially inbreed in older social groups, some categories of inbreeding appear more common than others. For example, we recorded eight instances of incest between fathers and daughters (of a possible 160 observations; electronic supplementary material, table S3) but none between mothers and sons (of a possible 170 observations), a highly significant difference (binomial proportions test, *χ*^2^ = 6.73, *p* = 0.0095). This may be because female banded mongooses begin breeding at one year but males rarely reproduce until they are three or four years old [8]. Young females may therefore have a high risk of encountering their fathers, while breeding males are unlikely to encounter their mothers, who have since died.

In other mammals where females are likely to encounter their father, females either disperse from their natal group prior to breeding or mate extra-group [2]. Although both of these strategies are effective at avoiding inbreeding in the banded mongoose, the majority of females mated within their natal group. Why, therefore, do not all females outbreed? Theory predicts that regular inbreeding may occur under circumstances where the costs of inbreeding are outweighed by the costs of inbreeding avoidance [6]. It is possible that banded mongooses may have particularly high costs of dispersal, as members of newly founded groups suffer an annual adult mortality rate (0.33) almost three times that of resident groups (0.12) [8]. Similarly, violent encounters between neighbouring groups mean that extra-group mating risks injury [8]. Hence, there might be a net benefit, at least to some females, of breeding within the natal group. Alternatively, inbreeding may be tolerated if the costs of inbreeding depression are relatively low. For example, (allo)parental investment towards inbred offspring could potentially buffer any fitness costs of inbreeding [3]. These possibilities will be the subject of future study.

How animals balance the costs of inbreeding and inbreeding avoidance is important to understand as this can be a fundamental determinant of patterns of dispersal, reproductive skew and cooperative interactions [5]. In the majority of cooperatively breeding vertebrates, the balance seems tipped towards inbreeding avoidance, at least at the within-group level. Identifying species where inbreeding is a normal part of the mating system will allow us to investigate how this balance can be reversed and to understand inbreeding in the context of cooperation and conflict within social groups.

## Supplementary Material

Supplementary material

## Supplementary Material

Data 21-11-14

## Data accessibility

Microsatellite sequences are available from Genbank: accession numbers AF271115, AF271117, AF271118, AF271120, AY142693, AY142694, AY142696, AY142697, AY142700, AY142703, AY155580, AY090498, EU045417, EU045419, FJ357430, FJ357432, FJ357438 and ERP000497. The life history and relatedness data used for analysis are available in the electronic supplementary material.
