# Supplementary material for: Evidence for frequent incest in a cooperatively breeding mammal
Source: Biol Lett. 2014 Dec;10(12):20140898. doi: 10.1098/rsbl.2014.0898 (PMC4298196; doi:10.1098/rsbl.2014.0898)
Supplement: Supplementary material [file rsbl20140898supp1.docx]

**Sample sizes for parentage analyses and statistical models**

Of the total of 1534 genotyped banded mongooses, we attempted to assign parentage to 1104 individuals. For the remaining 430 individuals, we did not have sufficient information on candidate parents to include them in the parentage analysis, usually because they were either born before the study began or were immigrants into the study area. Maternity was assigned at ≥ 95% confidence to 906 individuals (82% of those included in the maternity analysis). The 906 pups with maternity assigned at ≥ 95% confidence were included in the paternity analysis. Paternity was successfully assigned to 629 individuals at ≥ 95% confidence (69% of those included in the analysis). The lower success rate for assigning paternity in comparison to maternity is likely due to the higher number of candidate fathers (mean 72.5 candidate fathers per pup) in comparison to candidate mothers (mean 4.2 candidate mothers per pup). For 516 pups, the mother’s group of birth was known, allowing us to investigate whether maternal dispersal influenced inbreeding (Table S1). These 516 pups were produced by 258 unique breeding pairs (99 females and 121 males) from 12 social groups. When investigating whether inbreeding is more likely to occur in older or smaller groups (table S2), we used data from 167 communal litters from 14 groups. It was not necessary to restrict this model to cases where the mother’s group of birth was known as maternal dispersal is not included in this model. This model therefore included data from the 517 pups for which parentage was assigned at ≥ 95% confidence and which were not fathered by an extra-group male.

Table S1. A general linear mixed model (GLMM) investigating under what circumstances females conceived to related males. Breeding pair relatedness was fitted as the response variable and breeding situation (whether the female bred in her natal group with a resident male, in her natal group with an extra-group male, after dispersal with a resident male, or after dispersal with an extra-group male) was fitted as an explanatory factor (four-level factor). The identities of the mother, father, the group that the mother belonged to and the year in which she bred were included as random factors. P-values indicate where breeding pair relatedness was significantly different from zero. The model included 258 breeding pairs, comprising 99 females and 121 males from 12 social groups over 14 years. Two groups were excluded from the analysis as they formed before the study began so the origin of the females was not known. One further group was formed after the study began but it was possible to include a subset of females that were born after we began collecting data from the group.

| **Breeding situation** | **Estimate** | **Standard Error** | **t-value** | **p-value** |
| --- | --- | --- | --- | --- |
| Female bred in her natal group with a resident male | 0.133 | 0.024 | 5.47 | 1.08x10^-7^ |
| Female bred in her natal group with an extra-group male | -0.012 | 0.030 | -0.397 | 0.692 |
| Female bred after dispersal with a resident male | -0.069 | 0.039 | -1.78 | 0.077 |
| Female bred after dispersal with an extra-group male | -0.126 | 0.071 | -1.79 | 0.075 |

Table S2. A GLMM investigating whether inbreeding was more likely to occur in older or smaller groups. The mean relatedness of breeding pairs per communal litter was fitted as the response variable. Group age (the number of days since the group was founded), and group size at litter conception (the number of individuals over 1 year old present in the group 60 days before the litter was born) were included as explanatory variables. The year and group identity were fitted as random factors. For 3 groups, the exact year of formation was not known as the groups were founded before observations began. Here, the minimum age of the group was used. As extra-group paternity could mask inbreeding through reducing mean levels of relatedness between the parents of litter, we excluded cases of extra-group paternity from this analysis. Data included 167 communal litters from 14 groups across 15 years.

| **Variable** | **Estimate** | **Standard Error** | ***χ*^2^** | **p-value** |
| --- | --- | --- | --- | --- |
| Intercept | -0.041 | 0.046 |  |  |
| Group age | 0.000035 | 0.000015 | 6.23 | 0.013 |
| Group size | 0.0012 | 0.0024 | 0.25 | 0.62 |

Table S3. Frequency of the occurrence of each type of inbreeding event out of the total times a given event could have been detected from the pedigree data, along with the probabilities of these occurrences resulting from incorrect parentage assignments, given the Cervus confidence limits. In order to be conservative when calculating the probability of misassignment, we assumed that all assignments were made at 95% confidence (the minimum accepted confidence level). As the presence of unsampled individuals in the population can lead to an overestimation of inbreeding rates, we adjusted our calculations for an incomplete pedigree following Marshall et al (2002) [1].

| Category of inbreeding | Inbreeding coefficient (*f*) | Frequency | Percentage | Percentage adjusted for incomplete pedigree [1] | Maximum probability of one case resulting from mis-assignment | Maximum probability of all cases resulting from mis-assignments |
| --- | --- | --- | --- | --- | --- | --- |
| Father-Daughter | 0.25 | 8/160 | 5% | 5% | 0.26 | 0.00002 |
| Mother-Son | 0.25 | 0/170 | 0% | 0% | 0.26 | NA |
| Full Siblings | 0.25 | 3/81 | 3.7% | 3.15% | 0.37 | 0.05 |
| Half-siblings | 0.125 | 6/81 | 7.4% | 6.29% | 0.37 | 0.003 |
| Maternal Grandfather-granddaughter | 0.125 | 0/43 | 0% | 0% | 0.37 | NA |
| Paternal Grandfather-granddaughter | 0.125 | 0/22 | 0% | 0% | 0.37 | NA |
| Maternal Grandmother-Grandson | 0.125 | 0/76 | 0% | 0% | 0.37 | NA |
| Paternal Grandmother-Grandson | 0.125 | 0/35 | 0% | 0% | 0.37 | NA |
| Maternal Uncle-niece | 0.125 | 0/11 | 0% | 0% | 0.46 | NA |
| Paternal Uncle-niece | 0.125 | 1/10 | 10% | 8.5% | 0.46 | 0.46 |
| Maternal Aunt-nephew | 0.125 | 0/31 | 0% | 0% | 0.46 | NA |
| Paternal Aunt-nephew | 0.125 | 0/11 | 0% | 0% | 0.46 | NA |
| Double first cousin | 0.125 | 0/2 | 0% | 0% | 0.66 | NA |
| Other * | 0.125 > n > 0 | 12/123 | 9.8% | Variable | Variable | Variable |
| Total with close inbreeding | 0.25 | 11 | 8.5% | 8.0% |  |  |
| Total with moderate inbreeding | 0.125 | 7 | 16.7% | 14.3% |  |  |
| Total with close or moderate inbreeding | ≥ 0.125 | 18 | 23.8% | 21.1% |  |  |

* there were 123 pups with at least one set of great grandparents identified (the minimum required to detect low levels of inbreeding; *f*<0.125). In these cases, the probability of mis-assignment varies depending on the exact category of inbreeding event.

**References**

1. Marshall, T.C., Coltman, D.W., Pemberton, J.M., Slate, J., Spalton, J.A., Guinness, F.E., Smith, J.A., Pilkinton, J.G., Clutton-Brock, T.H. 2002 Estimating the prevalence of inbreeding from incomplete pedigrees. *Proceedings of the Royal Society of London Series B, Biological Sciences* **269**,1533-1539.
